# Behavioral intention to use distance teaching in the pandemic era

**DOI:** 10.3389/fpsyg.2022.879316

**Published:** 2022-08-11

**Authors:** Chih-Hung Tseng, Ching-Tang Wang, Chin-Hsien Hsu, Jing-Wei Liu

**Affiliations:** ^1^Department of Leisure Industry Management, National Chin-Yi University of Technology, Taichung, Taiwan; ^2^Department of Industrial Engineering and Management, National Taipei University of Technology, Taipei, Taiwan; ^3^Department of Sport Information and Communication, National Taiwan University of Sport, Taichung, Taiwan

**Keywords:** distance teaching, technology acceptance model, information system success model, diffusion of innovations theory, structural equation model

## Abstract

This study aimed at exploring the impact of post-epidemic era on teachers’ behavioral intention of distance education. In this study, purposive sampling method was used to enroll 390 teachers in colleges and universities, high schools and vocational schools, and junior high and elementary schools to be the research subjects for the questionnaire survey. A total of 360 questionnaires were collected for statistics, and AMOS 23.0 statistical software was used to analyze the correlation between variables. Meanwhile, a structural equation model was used to analyze and verify the impact of the technology acceptance model, information system success model, and diffusion of innovations theory. According to the results, the system quality of distance teaching platforms certainly affected perceived usefulness, and perceived usefulness affected teachers’ attitudes toward using distance teaching systems. In addition, perceived ease of use had an impact on perceived usefulness, and trialability had an impact on teachers’ attitudes toward using distance teaching systems. Finally, teachers’ attitude toward using a distance teaching system also affected their behavioral intention. According to the results, suggestions were made in this study to teachers and students for distance teaching. Additionally, the main contribution of this study lies in providing specific strategies for further distance teaching through empirical results in combination with on-site teaching.

## Introduction

The outbreak of the COVID-19 epidemic at the end of 2019 not only spread rapidly in the Asia-Pacific region, but many countries around the world began to feel the impact of the virus in early 2020. It gradually turned into a global pandemic that some people consider a long-term war between humans and the virus. Ko (2021) pointed out that the spread of the epidemic has had a huge impact on global aviation, tourism, entertainment, sports, oil markets, and financial markets and has been described as the most severe crisis facing the world since World War Two by many international organizations and media as well as the worst public health incident in history that has severely hit the global economy and tested the resilience of world leaders and companies.

Taiwan has also been affected by this wave of the epidemic. According to data of Centers for Disease Control, Ministry of Health and Welfare (Taiwan) in 2021, as of November 24, 2021, there are currently 257,659,504 confirmed cases worldwide and 5,172,695 cumulative deaths, including 16,544 confirmed cases and 848 deaths in Taiwan, and the global fatality rate is 2.01% (Centers for Disease Control, Ministry of Health and Welfare 2021). With the spread of the epidemic, the barriers between people under social distancing as well as the unease and anxiety caused by the ubiquitous virus are growing. With the announcement from the Centers for Disease Control, Ministry of Health and Welfare (2021) on May 19 that the national epidemic alert will be raised to Level 3 from then until May 28, the impact on all walks of life soon expanded.

The Ministry of Education continually reminds schools at all levels to be prepared for epidemic prevention. At the same time, in order to ensure that students’ learning is not interrupted, distance teaching has become one of the ways to solve the problem that students cannot go to the classroom in person under the impact of the epidemic. In fact, teachers and students have now become more familiar with traditional distance teaching. Lo and Chen (2021) pointed out that distance teaching was practiced for a long time before the outbreak of the epidemic, but in the past, the premise of distance teaching research was mostly that students consciously chose this learning mode when both teachers and students were well informed and prepared. Unlike traditional distance teaching, distance teaching in the post-epidemic era means that relevant units of the Ministry of Education or private digital learning resources provide websites and platforms for the teachers and students at all levels of schools to successfully carry out distance teaching.

With the implementation of distance teaching under the epidemic, some problems have gradually emerged. For example, Cheng and Lee (2021) presented several problems in distance teaching. For teachers: (1) teacher-student interaction is relatively difficult; (2) the willingness of distance teaching is not high; (3) the teaching preparation time becomes longer; and (4) the fairness of online assessment is questioned. For students: (1) when there is a problem with online self-learning, the answer may not be available immediately; (2) one may miss the class time when synchronizing online courses; (3) it may be easier to get distracted from studying in a non-school learning field; and (4) the infrastructure for online learning may be insufficient. Due to the above problems, it is worthy to explore whether the distance teaching system is appropriate as a teaching medium in the post-epidemic ear. Therefore, this study used the Technology Acceptance Model proposed by Davis (1989) in combination with the Information System Successful Model proposed by DeLone and McLean (1992) to establish a framework with teachers as the research subjects. One motivation of this study is to further discuss the influence of the system quality of the distance teaching platform, information quality, and service quality on perceived ease of use and perceived usefulness.

A teacher’s lack of digital competence and differences in hardware equipment may also cause the teaching impact to be less than expected, such as teachers having limited basic computer skills, teachers being unfamiliar with the distance teaching software used by the school, or the teaching process being interrupted by insufficient network bandwidth. At the same time, to uphold the original intention of teaching, teachers pay more attention to the use of the distance teaching system for the purpose of improving teaching effectiveness. It thus deserves further investigation of whether the characteristics of the distance teaching system will affect a teacher’s attitude toward the use of the system. Therefore, the second research motivation is to explore the impact of teachers’ perceived usefulness and perceived ease of use on their attitudes toward using distance learning systems.

The Technology Acceptance Model and Information System Success Model are often used to discuss how users use a certain system (Lin, 2018; Sun and Chen, 2021). The Diffusion of Innovations Theory is mostly employed to explore a user’s cognition and perception of the information quality and system quality of the information system, which will affect the operation and use of the system (Gharaibeh et al., 2020; Pinho et al., 2020; Yang and Lin, 2020). It is also found from the relevant literature that the topics for research and discussion of distance teaching under the COVID-19 epidemic are diversified. For example, with the metacognition theory Ko (2021) noted the issues related to the process of autonomous learning when students and teachers faced online teaching. Huang (2021) explored the issue of learning satisfaction caused by distance learning under the COVID-19 epidemic and pointed out that interaction and feedback have a significant positive impact on learning satisfaction. Fang (2021) discussed the effectiveness, problems, and reflections of the first Chinese language center in Taiwan to implement emergency online courses due to COVID-19 from the administrative perspective. Fang also pointed out that teachers and students believe that the biggest problem of online classes can be classified as unstable network speed and quality, noise interference in class, and teachers’ insufficient online teaching ability. From the above-mentioned related literature, it can be seen that the related discourses on behavioral intentions of distance teaching systems are relatively scarce. Therefore, the third motivation of this study is to examine the influence of teachers’ attitudes toward distance teaching systems on their behavioral intentions.

## Literature review

### Technology acceptance model

The Technology Acceptance Model is often regarded as an appropriate theoretical framework for discussing the issues of people’s use behavior of technology-related services or equipment (Byun et al., 2018; Min et al., 2019). Based on the Theory of Reasoned Action by Fishbein and Ajzen (1975), Davis et al. (1989) developed the Technology Acceptance Model (TAM), which includes 5 main aspects: perceived usefulness, perceived ease of use, attitude of use, intention to use, and use behavior. Among them, perceived usefulness is the degree to which users subjectively believe that the use of a specific information system can improve the effectiveness of their present or future work, while perceived ease of use refers to the degree to which users subjectively believe that they can save effort when using a specific information system (Davis, 1989).

In the case of this article, perceived usefulness can refer to the degree to which students use distance teaching systems to improve learning effectiveness under the epidemic situation, whereas perceived ease of use can indicate whether students are operating the distance teaching model system smoothly. Lee and Fang (2013) also held a similar view; they suggested that consumers’ acceptance and use of information technology are prerequisites for the information technology to fully exert its expected benefits. As can be seen from the above-mentioned literature, the TAM theory emphasizes that the behavior of individuals is based on their own inner wishes. When individuals decide to take on a certain behavior, they will give priority to the hidden meaning behind their behavior.

The shortcomings of this TAM theory have gradually been proposed as it becomes applied to more and more studies. For example, Venkatesh and Davis (2000) found in many studies that TAM cannot fully express its meaning, and its interpretive ability is limited in terms of use intention and use behavior. Therefore, the original TAM was extended and the second generation of TAM theory was proposed, in which the social influence process covers subjective norms, voluntariness, image, and experience, while auxiliary cognitive processes include work relevance, output quality, and result display. Rather than exclude subjective norms from the TAM theory by Davis et al. (1989), as they pointed out that subjective norms have little impact on TAM and are not conducive to explaining and predicting actual behavior, the second-generation TAM theory points out that subjective norms achieve a direct impact on the perceived usefulness in the process of social influence *via* the two means of internalization and identification, thereby indirectly acting on the intention to use. Through these two impacts, the user will strengthen belief in the person who he or she thinks is very important to his or her own belief, thereby significantly increasing the identification of perceived usefulness.

### Information system success model

With the establishment of information systems, the performance presented by the information system itself has gradually attracted attention. This is the background of the construction of successful model information systems. By integrating “communication theory” and “information impact theory,” DeLone and McLean (1992) summarized six measurement indicators to develop the “Information System Success Model.” The six measures are explained as follows.

1. System Quality: In the network environment, “System Quality” measures the quality characteristics required by the information system itself.
2. Information Quality: “Information Quality” is used to measure the quality of the information content output by the information system.
3. Use: “Use” is a measure of the user’s use of the information system, including actual use and perceived use.
4. User Satisfaction: “User Satisfaction” is the user’s response to the output of the information system.
5. Individual Impact: “Individual Impact” refers to the impact of the information system on the user’s personal behavior.
6. Organizational Impact: “Organizational Impact” is the impact of the information system on organizational performance.

It can be seen from the above content that the three levels of the Information System Success Model can be used as the basis for the arguments when assessing whether the information system is successfully applied and for achieving the expected efficacy and goals. In view of the high demand for service quality, DeLone and McLean (2003) compiled relevant research, revised the original Information System Success Model to take into account the service quality aspects proposed by Pitt et al. (1995), and revised and updated the individual and organizational impacts. The revised Information System Success Model is mainly a change in degree, rather than a physical change. In the revised Information System Success Model, the concept of “intention to use” has been added to the original variable of “use,” and “individual impact” and “organizational impact” have been combined into “net benefit.” Therefore, the revised Information System Success Model divides the information system into the six conceptual indicators of “system quality,” “information quality,” “service quality,” “system use intention and use,” “user satisfaction,” and “net benefit.” Among them, “net benefit” is the most important factor for the success of an information system, and it refers to all the positive and negative impacts of the e-commerce system on customers, suppliers, enterprises, markets, industries, the economy, and society. As noted above, today’s distance teaching does have some problems. Therefore, understanding the value perception evaluation and the students’ attitude of use toward the distance teaching system is crucial to the decision on their intention to continue to use the information system. Related research also indicates the feasibility of using the Information System Success Model to explore the behavioral intentions of users for digital learning or distance learning systems (Mo and Chen, 2018; Kuo, 2019; Kao, 2020).

### Diffusion of innovations theory

When new things or a topic of how new technologies are promoted and disseminated are under discussion, the focus of attention turns to understanding the attributes and characteristics of the people accepting innovations - that is, how cognition affects the process of their decision to adopt. Indeed, the Diffusion of Innovations Theory is the theoretical basis of the argument. As Lai (2010) mentioned, the Diffusion of Innovations Theory was first applied to the promotion of pesticides and the diffusion of new drugs, and it was subsequently extended to different fields such as marketing management, sociology, education research, public health and medical sociology, and economics. At the same time, it also has reached into the fields of mass communication, communication management, journalism, information communication, publishing business management, and business management. The common point is that the research content relates to the dissemination of new technologies or things.

Rogers (2010) pointed out that the so-called diffusion of innovations is defined as a process in which an innovation is spread among certain members of the social system through a specific communication channel. Greenhalgh et al. (2004) mentioned that so-called diffusion is possibly a planned promotion. For example, a planned healthy living strategy is applied to a chronic disease prevention and control plan, or it may be an accidental promotion formed without a plan. As learned from the above description, when people recognize that a thing or technology is a new invention or has not been used, and it is planned or spread among people occasionally at the same time, then it can be said to be an innovation.

Rogers (2010) further noted that the process of spreading new things is divided into five stages: knowledge, persuasion, decision, implementation, and confirmation. This describes a process in which people or decision-making organizations will have a considerable degree of understanding of the innovation once they know the existence of a certain innovation. They then will develop a good impression of this innovation or are willing to accept it in attitude through the persuasion of others, face the choice of accepting or rejecting the innovation, and take practical actions to accept or reject the innovation. The knowledge stage is when the individual starts to collect relevant information after learning of the innovation plan. The persuasion stage is for the individual to form an attitude of approval or rejection of the innovative plan after the integration of knowledge. The decision stage is for the individual to make a decision on accepting or rejecting the innovation plan. The implementation stage is the individual’s specific implementation of the decision on the innovation plan (to accept or reject it). The confirmation stage refers to the individual seeking confirmation of the previously implemented decision and examining whether to continue to accept or reject the original innovation that has been accepted or rejected.

For the purpose of this article, the knowledge stage is when students receive the new distance learning software, they begin to learn how to use it. The persuasion stage is showing an attitude toward the software according to the process of their own use. In the decision stage, the students decide to use the software after learning to use the software. The implementation stage is for the students to accept the distance teaching software. Finally, in the confirmation stage, the software is used as an online learning tool. Its acceptance factor may be that students personally feel that the software is easy to use, or students have limited choices offered by the school.

Rogers (2010) constructed five characteristics of innovative things as follows. (1) Relative advantages: the degree of advantage that the new thing has for the replaced old one. If the individual perceives more advantages, then his or her adoption of the new thing will be faster. (2) Compatibility: the degree that the new thing meets the needs of the potential acceptor. The higher the degree of compatibility is, the less the individual needs to change his or her habits to cooperate with the introduction of innovation, the lower is the uncertainty for the potential acceptor, and the faster is the adoption of new things. (3) Complexity: the more complicated new things will reduce the speed of their adoption or stop their diffusion. (4) Trialability: whether new things can be inspected or tried. Innovations that can be inspected or tried can reduce uncertainty and increase the recipient’s adoption of innovative things. (5) Observability: the degree to which the results of new things can be observed by others. If the introduction of the new thing is easy to be observed by others, or it can be evaluated by others’ retelling, use, and promotion of it, then the speed of its adoption will also accelerate. It can be seen from the relevant literature that the Diffusion of Innovations Theory is widely used in education-related fields (Buc and Divjak, 2015; Hussain, 2017; Sasaki, 2018). Therefore, this research also used the Diffusion of Innovations Theory to further explore the behavioral intentions of students using distance learning.

### Relationship between variables

Confirming the relationship between variables is the basis for constructing the research hypothesis and research model of this article. In the model constructed in this study, the relationship between variables was empirically confirmed by related research: information quality has a positive impact on perceived usefulness (Chen, 2009; Machda, 2016; Kim et al., 2019; Nirwanto and Andarwati, 2019; Tiana et al., 2019), information quality has a positive impact on perceived ease of use (Chen, 2009; Machda, 2016), system quality has a positive impact on perceived usefulness (Kim et al., 2019; Nirwanto and Andarwati, 2019), system quality has a positive impact on perceived ease of use (Ahn et al., 2007; Chen, 2009; Tumenbayar and Navchaa, 2019), service quality has a positive impact on perceived usefulness (Chen, 2009), service quality has a positive impact on perceived ease of use (Ahn et al., 2007; Chen, 2009), perceived ease of use has a positive impact on perceived usefulness (Machda, 2016), perceived usefulness has a positive impact on attitude of use (Xie, 2016; Hsiung, 2021), perceived ease of use has a positive impact on attitude of use (Hsiung, 2021), relative advantages have a positive impact on attitude of use (Chen, 2009; Kuo, 2015), compatibility has a positive impact on attitude of use (Tsai, 2013), trialability has a positive impact on attitude of use (Xie, 2016; Chang, 2019), observability has a positive impact on attitude of use (Chang, 2019), and attitude of use has a positive impact on behavioral intention (Xie, 2016; Chang, 2019).

## Research method

### Research structure

**Figure 1:**
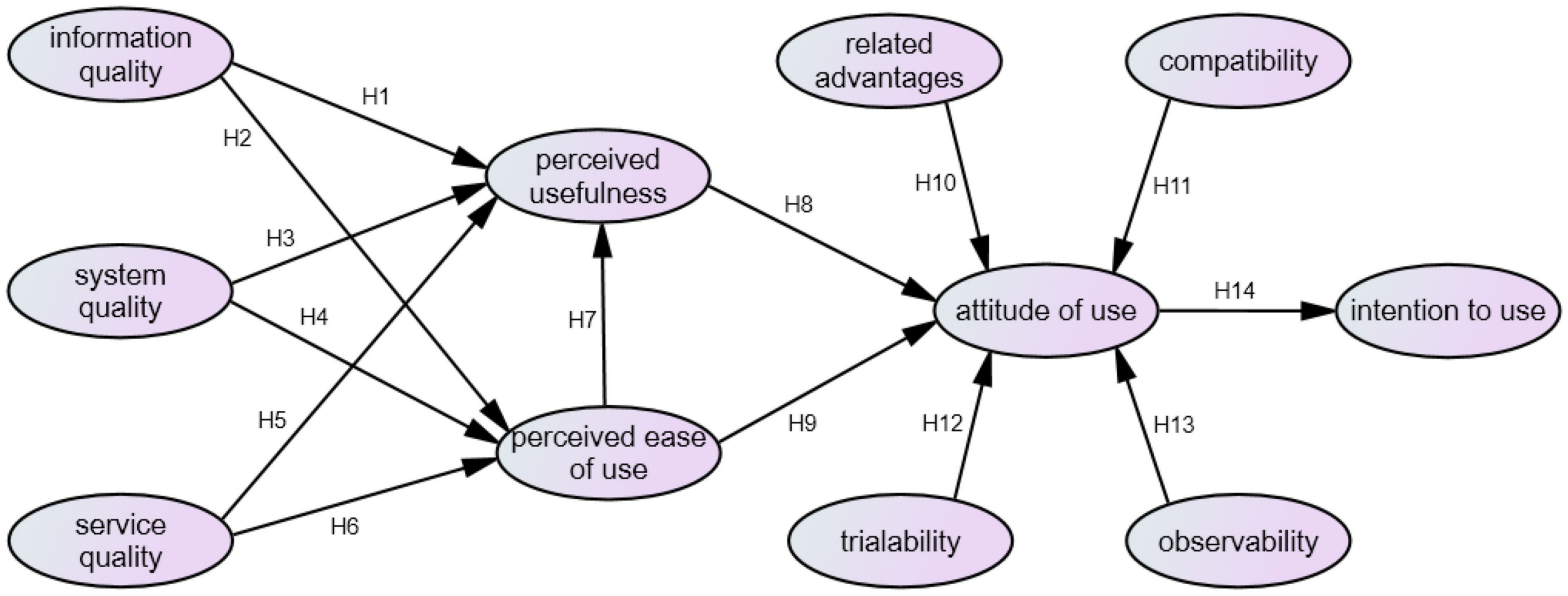
Research structure.

This study empirically explored the impact of the intention of adopting distance teaching behavior in the post-epidemic era. According to the research purpose and related literature, the research structure proposed is shown in Figure 1.

### Research hypotheses

The following 14 research hypotheses were put forward by this study based on the research purposes and related literature.

*H1*: Information quality has a significant impact on perceived usefulness.

*H2*: Information quality has a significant impact on perceived ease of use.

*H3*: System quality has a significant impact on perceived usefulness.

*H4*: System quality has a significant impact on perceived ease of use.

*H5*: Service quality has a significant impact on perceived usefulness.

*H6*: Service quality has a significant impact on perceived ease of use.

*H7*: Perceived ease of use has a significant impact on perceived usefulness.

*H8*: Perceived usefulness has a significant impact on attitude of use.

*H9*: Perceived ease of use has a significant impact on attitude of use.

*H10*: Related advantages have a significant impact on attitude of use.

*H11*: Compatibility has a significant impact on attitude of use.

*H12*: Trialability has a significant impact on attitude of use.

*H13*: Observability has a significant impact on attitude of use.

*H14*: Attitude of use has a significant impact on intention to use.

### Research subjects

This study empirically explored the impact of the intention of adopting distance teaching behavior in the post-epidemic era. The teachers from colleges, high schools and vocational schools, junior high schools, and elementary schools were taken as the research subjects. In total, 390 teachers were selected as the research samples to conduct a questionnaire survey, 360 questionnaires were recovered for a recovery rate of 92%, and the number of valid questionnaires was 329 for a valid questionnaire rate of 91%.

### Research tools

The content of this research questionnaire was revised and complied mainly by referring to the relevant literature of questionnaires by Davis (1989), Moore and Benbasat (1991), Premkumar and Potter (1995), Taylor and Todd (1995), Bhattacherjee (2001), Cyr et al. (2006), Roca et al. (2006), Ahn et al. (2007), Walczuch et al. (2007), Jeong (2011), Chang (2019), and Pan (2021). The questionnaire was divided into four parts with a total of 41 items. The Likert five-point scale was used in this study, and each item option was given a score of 1 to 5 from “Strongly disagree” to “Strongly agree.”

### Data processing and analysis

In this study, the questionnaires were collected, and invalid questionnaires were eliminated according to the criteria of valid questionnaires. The data were filed with SPSS 23.0 statistical software, and the correlation between variables was analyzed with AMOS 23.0 statistical software.

## Research results

### Sample characteristics

This study took teachers from colleges and universities, high schools, vocational schools, and junior high and elementary schools as the test samples. There were a total of 329 valid samples. Among them, in terms of gender, there were 224 males, accounting for 68.1% of the valid samples, and 105 females, accounting for 31.9% of the valid samples. In terms of age, there was a total of 124 people aged 46–55, accounting for 37.7% of the valid samples, and 4 people over 66 years old, accounting for 1.2% of the valid samples. For educational level, most of them (a total of 198 people) had a master’s degree, accounting for 60.2% of the valid samples, while the least of them (5 people) had a junior college degree or less, accounting for 1.5% of the valid samples. In terms of working experience, there was a total of 159 people with more than 21 years of working experience, accounting for 48.3% of the valid samples, and only 4 people with working experience under 1 year, accounting for 1.2% of the valid samples. In regards to the type of school, most of them (167) were in colleges and universities, accounting for 50.8% of the valid samples, and only 27 people were in elementary school or below, accounting for 8.2% of the valid samples, Regarding school location, 208 people were in the central region, accounting for 63.2% of the valid samples, and the least number of subjects (two people) was in the outlying islands, accounting for 0.6% of the valid samples. In terms of the most frequently used video software, Google Meet had a maximum of 236 users, which accounted for 71.7% of the valid samples, while Cisco Webex and Skype had the least number of users at one person each, accounting for 0.3% of the valid samples. The details are shown in Table 1.

**Table 1:** Subjects’ characteristics.

Variable	Category	Number of times	Percentage %	Cumulative percentage %
Gender	Male	224	68.1	68.1
	Female	105	31.9	100.0
Age	Below 25 years old	5	1.5	1.5
	26–35 years old	37	11.2	12.8
	36–45 years old	91	27.7	40.4
	46–55 years old	124	37.7	78.1
	56–65 years old	68	20.7	98.8
	66 years old or older	4	1.2	100.0
Educational level	Doctor	84	25.5	25.5
	Master	198	60.2	85.7
	Undergraduate	42	12.8	98.5
	Junior college	3	0.9	99.4
	High/vocational school or below	2	0.6	100.0
Working experience	Less than 1 year	4	1.2	1.2
	1–5 years	25	7.6	8.8
	6–10 years	32	9.7	18.5
	11–15 years	42	12.8	31.3
	16–20 years	67	20.4	51.7
	21 years or more	159	48.3	100.0
Type of school	College and university	167	50.8	50.8
	High/vocational school	90	27.4	78.1
	Junior high school	45	13.7	91.8
	Elementary school	23	7.0	98.8
	Kindergarten	1	0.3	99.1
	Others	3	0.9	100.0
School location	North	80	24.3	24.3
	Central	208	63.2	87.5
	South	33	10.0	97.6
	East	6	1.8	99.4
	Outlying islands	2	0.6	100.0
The most frequently used video software	Microsoft Teams	37	11.2	11.2
	Google Meet	236	71.7	83.0
	Zoom	11	3.3	86.3
	Cisco Webex	1	0.3	86.6
	Skype	1	0.3	86.9
	Line	41	12.5	99.4
	Others	2	0.6	100.0

### Measurement model analysis

This study adopted CFA to verify the reliability and validity of the questionnaire, and item corrections were made by referring to the Modification Indices (MI; Chen, 2007). B1 and B13 of the Technology Acceptance Model Scale, C5 and C9 of the Information System Success Model Scale, and D6, D12, and other items of the Diffusion of Innovations Theory Scale were deleted in this study.

#### Test of convergent validity

Bagozzi and Yi (1988) pointed out that the composite reliability (CR) and average variance extracted (AVE) of the questionnaire dimensions should be measured in the convergent validity test. For good convergent validity of a questionnaire, it is recommended that the CR value should be greater than 0.6 and the AVE value should be greater than 0.5 (Fornell and Larcker, 1981). In this study, the convergent validity test was conducted for the Technology Acceptance Model, the Information System Success Model, and the Diffusion of Innovations Theory. The factor loadings of all the dimensions were between 0.65 and 0.90, the CR values were between 0.79 and 0.88, and the AVE values were between 0.57 and 0.76, conforming to the convergent validity standard recommended by Fornell and Larcker (1981), Bagozzi and Yi (1988), and Hair et al. (1998). This means the convergent validity of the questionnaire in this study is good, as shown in Tables 2–4.

**Table 2:** Technology acceptance model - confirmatory factor analysis.

Dimension	Indicator	Standardized load	Non-standardized load	S.E.	C.R. (t-value)	*p*	SMC	C.R.	AVE
Perceived ease of use	B2	0.72	1.00				0.52	0.86	0.68
	B3	0.90	1.08	0.07	14.84	***	0.82		
	B4	0.85	1.05	0.07	14.50	***	0.72		
Perceived usefulness	B5	0.81	1.00				0.66	0.88	0.65
	B6	0.87	1.05	0.06	18.55	***	0.75		
	B7	0.80	1.00	0.06	15.33	***	0.63		
	B8	0.76	0.92	0.06	14.30	***	0.58		
Attitude of use	B9	0.80	1.00				0.64	0.87	0.63
	B10	0.85	1.10	0.06	17.43		0.72		
	B11	0.73	0.83	0.06	14.01	***	0.53		
	B12	0.80	0.99	0.06	15.92	***	0.64		
Intention to use	B14	0.79	1.00				0.62	0.87	0.64
	B15	0.84	1.11	0.07	16.00	***	0.70		
	B16	0.81	0.90	0.06	16.00	***	0.66		
	B17	0.76	0.94	0.07	14.40	***	0.58		

***p < 0.001.

**Table 3:** Information system success model - confirmatory factor analysis.

Dimension	Indicator	Standardized load	Non-standardized load	S.E.	C.R. (t-value)	*p*	SMC	C.R.	AVE
Information quality	C1	0.84	1.00				0.70	0.87	0.62
	C2	0.85	1.10	0.06	17.83	***	0.72		
	C3	0.72	0.95	0.07	14.18	***	0.52		
	C4	0.75	0.92	0.06	14.84	***	0.56		
System quality	C6	0.86	1.00				0.73	0.88	0.72
	C7	0.87	1.05	0.05	19.17	***	0.75		
	C8	0.82	0.85	0.05	17.29	***	0.68		
Service quality	C10	0.76	1.00				0.58	0.79	0.57
	C11	0.85	1.09	0.07	14.94	***	0.73		
	C12	0.65	0.87	0.08	11.45	***	0.42		

***p < 0.001.

**Table 4:** Diffusion of innovations theory - confirmatory factor analysis.

Dimension	Indicator	Standardized load	Non-standardized load	S.E.	C.R. (t-value)	*p*	SMC	C.R.	AVE
Relative advantages	D1	0.87	1.00				0.76	0.87	0.69
	D2	0.87	0.99	0.05	18.95	***	0.76		
	D3	0.75	0.84	0.05	15.74	***	0.56		
Compatibility	D4	0.90	1.00				0.82	0.86	0.76
	D5	0.85	0.89	0.05	17.00	***	0.72		
Trialability	D7	0.72	1.00				0.52	0.80	0.57
	D8	0.81	1.06	0.08	13.31	***	0.66		
	D9	0.74	1.06	0.09	11.32	***	0.55		
Observability	D10	0.86	1.00				0.73	0.82	0.70
	D11	0.82	0.88	0.07	12.39	***	0.68		

***p < 0.001.

#### Discriminant validity

The confidence interval method (Bootstrap) was adopted for the discriminant validity test of this study. First, the Pearson correlation coefficient confidence interval among the questionnaire dimensions of this study was checked to see whether there was a value of 1 to determine whether the questionnaire dimensions were completely correlated. The results showed that the confidence interval between the questionnaire dimensions did not contain the value of 1, indicating that the questionnaire dimensions of this study have significant discriminant validity (Torkzadeh et al., 2003), as shown in Tables 5–7.

**Table 5:** Technology acceptance model - bootstrap correlation coefficient 95% confidence interval.

Parameters			Estimate	Bias-corrected		Percentile method	
				Lower bound	Upper bound	Lower bound	Upper bound
Perceived ease of use	↔	Perceived usefulness	0.53	0.42	0.62	0.42	0.63
Perceived ease of use	↔	Attitude of use	0.53	0.41	0.63	0.41	0.63
Perceived ease of use	↔	Intention to use	0.54	0.41	0.63	0.42	0.64
Perceived usefulness	↔	Attitude of use	0.84	0.78	0.89	0.78	0.89
Perceived usefulness	↔	Intention to use	0.65	0.57	0.72	0.56	0.72
Attitude of use	↔	Intention to use	0.80	0.71	0.87	0.71	0.87

Compiled by this study.

**Table 6:** Information system success model - bootstrap correlation coefficient 95% confidence interval.

Parameters			Estimate	Bias-corrected		Percentile method	
				Lower bound	Upper bound	Lower bound	Upper bound
Information quality	↔	System quality	0.52	0.39	0.64	0.40	0.64
Information quality	↔	Service quality	0.80	0.71	0.88	0.71	0.88
System quality	↔	Service quality	0.74	0.63	0.84	0.64	0.85

Compiled by this study.

**Table 7:** Diffusion of innovations theory - bootstrap correlation coefficient 95% confidence interval.

Parameters			Estimate	Bias-corrected		Percentile method	
				Lower bound	Upper bound	Lower bound	Upper bound
Relative advantages	↔	Compatibility	0.69	0.60	0.78	0.60	0.78
Relative advantages	↔	Trialability	0.57	0.44	0.66	0.44	0.67
Relative advantages	↔	Observability	0.43	0.28	0.55	0.29	0.56
Compatibility	↔	Trialability	0.67	0.58	0.76	0.58	0.76
Compatibility	↔	Observability	0.42	0.30	0.53	0.30	0.53
Trialability	↔	Observability	0.69	0.55	0.79	0.55	0.80

Compiled by this study.

#### Structural equation model analysis

The overall model fit of this study was tested with reference to the structural model analysis of Hair et al. (1998), and the seven indicators of chi-square value (*χ*^2^), the ratio of *χ*^2^ to its degrees of freedom, GFI, AGFI, RMSEA, CFI, and PCFI were used for the test of the overall model fit. Bagozzi and Yi (1988) indicated that a smaller ratio of *χ*^2^ to its degrees of freedom is better, and the revised ratio of this study was 2.58. Hair et al. (1998) stated that the GFI value and the AGFI value being closer to 1 are better, and the corrected GFI and AGFI for this study were 0.82 and 0.80, respectively. Browne and Cudeck (1993) argued that the best RMSEA value should be less than 0.08, and the RMSEA value in this study was corrected to 0.07. The CFI standard value should be greater than 0.90, and the CFI value was corrected in this study to 0.90. The PCFI value must be at least greater than 0.50, and the revised PCFI of this study was 0.80. These findings show that the overall fit indices of the research results have reached the criteria, as seen in Table 8.

**Table 8:** Overall Model Fit Analysis.

Goodness of fit index	Allowable range	Revised model	Model fit determination
*χ*^2^(Chi-square)	The smaller, the better	1383.45	
Ratio of *χ*^2^ to the degree of freedom	<3	2.58	Pass
GFI	>0.80	0.82	Pass
AGFI	>0.80	0.80	Pass
RMSEA	<0.08	0.07	Pass
CFI	>0.90	0.90	Pass
PCFI	>0.50	0.80	Pass

**Figure 2:**
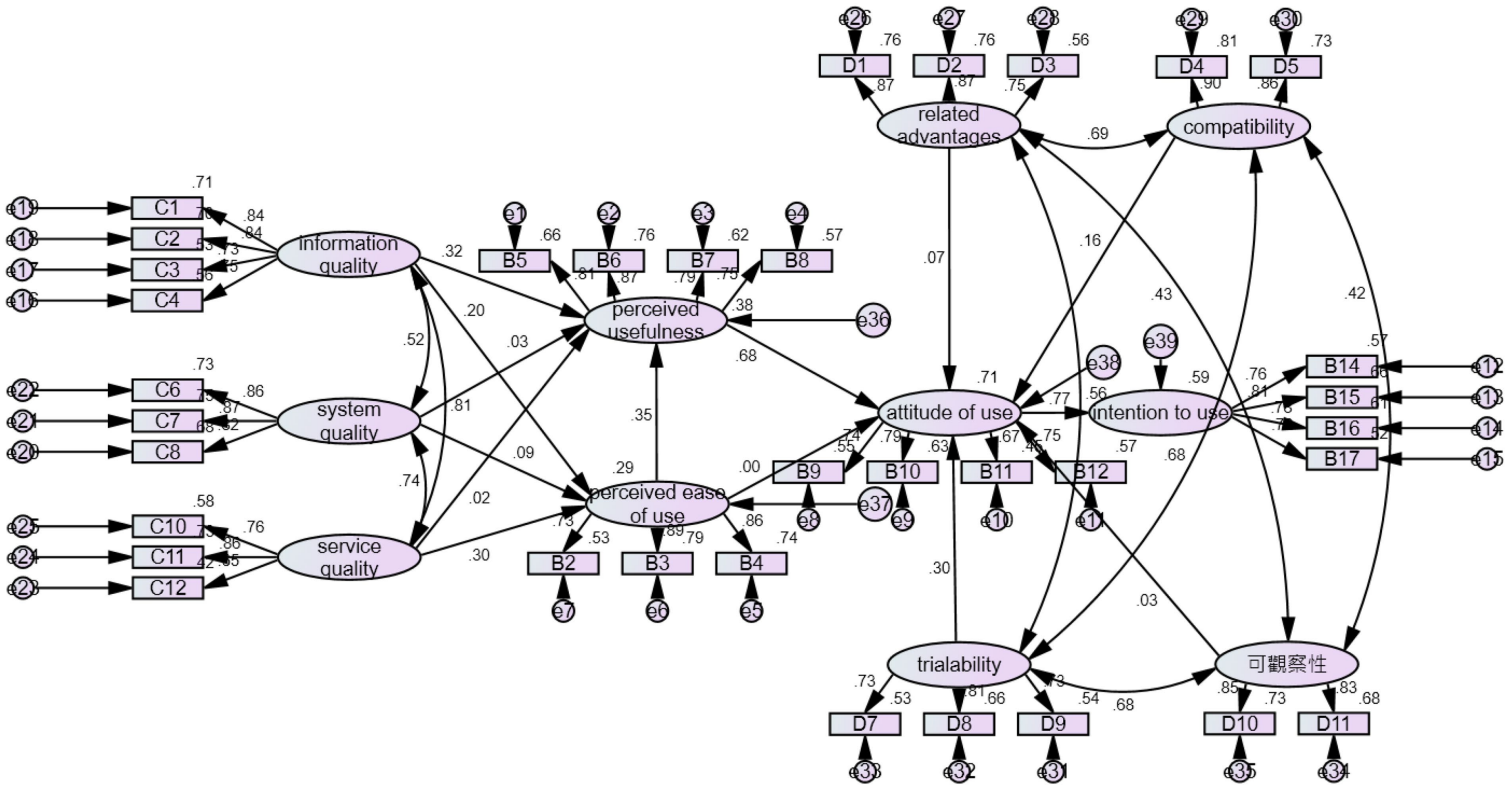
Empirical research model of behavioral intentions influencing adoption of distance teaching in Post-epidemic Era.

In the results of this study shown in Figure 2 and Table 9, five hypotheses were established. Among them, information quality has a positive impact on perceived usefulness. The result of this study is the same as that of Kim et al. (2019), and the possible reason is the better is the quality of the teaching-related content output from the distance teaching system, the greater is the degree of students’ use of the distance teaching system to improve the learning effectiveness. In addition, this study found that perceived ease of use has a positive impact on perceived usefulness, and the result of this study is similar to the research result of Machda (2016). The possible reason is that the smoother is the operation related to the distance teaching mode system, the greater is the degree of students’ use of the distance teaching system to improve their learning effectiveness. On the other hand, this study showed that perceived usefulness has a positive impact on attitude of use. The result of this study is similar to the research conclusion of Hsiung (2021). This may be due to the higher the degree of improving the students’ learning effectiveness *via* using the distance learning system is, the more likely they are to have a positive attitude toward using distance learning. This study pointed out that trialability has a positive impact on attitude of use. The result of this study is similar to the research result of Chang (2019). This is probably because a school provides many opportunities for practice in order for the students to get familiar with the operation interfaces and use methods so that students are familiar with the system and have a positive view of distance teaching. Finally, this study revealed that the use of attitude has a positive impact on behavioral intentions, and the result of this study is in line with that of Xie (2016). The possible reason is that when students have an attitude of accepting distance teaching, their intention to use the distance teaching system is also higher.

**Table 9:** Empirical results of research hypotheses.

Hypothesis	Path relationship	Path value	Hypothesis established or not
1	Information quality has a significant impact on perceived usefulness	0.32*	Established
2	Information quality has a significant impact on perceived ease of use	0.20	Not established
3	System quality has a significant impact on perceived usefulness	0.03	Not established
4	System quality has a significant impact on perceived ease of use	0.09	Not established
5	Service quality has a significant impact on perceived usefulness	0.02	Not established
6	Service quality has a significant impact on perceived ease of use	0.30	Not established
7	Perceived ease of use has a significant impact on perceived usefulness	0.35^*^	Established
8	Perceived usefulness has a significant impact on attitude of use	0.68*	Established
9	Perceived ease of use has a significant impact on attitude of use	0.00	Not established
10	Related advantages have a significant impact on attitude of use	0.07	Not established
11	Compatibility has a significant impact on attitude of use	0.16	Not established
12	Trialability has a significant impact on attitude of use	0.30^*^	Established
13	Observability has a significant impact on attitude of use	0.03	Not established
14	Attitude of use has a significant impact on intention to use	0.77^*^	Established

* *p* < 0.05.

## Conclusion and suggestions

Through empirical analysis, according to the results of this study, the research objectives discussed in the introduction were verified, system quality of the distance teaching platform certainly affected perceived usefulness, and perceived usefulness affected teachers’ attitude of using distance teaching system. Furthermore, perceived ease of use had impact on perceived usefulness, and trialability had impact on teachers’ attitude of using distance teaching system. Finally, teachers’ attitude of using distance teaching system also affected their behavioral intention.

Based on the above research results, several suggestions are put forward by this study for reference.

The results of this study indicate that information quality has a significant impact on perceived usefulness. Therefore, it is recommended when teachers implement distance teaching that relevant class data or information must be clearly communicated in addition to clear course materials. For example, some teachers forget to turn off the microphone during the lecture, which causes the students to be unable to hear the content of the audio and video when playing the audio-visual teaching materials, thereby reducing information quality, preventing the students from concentrating on listening to the content of the course, and making them distracted and wanting to leave the distance teaching screen. If the teacher fails to communicate clearly enough in determining the feedback method for students or the time limit for uploading homework, then it will result in students not being able to submit their homework or deliver timely feedback, thus lowering the expectation of improving learning effectiveness from the distance teaching system. In addition, most teachers have less experience in distance teaching and are not used to teaching in front of the camera. Therefore, the teaching process is more unnatural and affects the interaction between students and teachers to some extent. It is recommended that teachers can practice more tips on teaching in front of the camera after class, and relevant units of the school can also handle distance teaching research so that teachers can master more online teaching skills and strive to be smoother in the delivery of the course.

This study also found that trialability has a significant impact on attitude of use. Therefore, it is recommended that relevant schools can select appropriate students as seed teachers and provide training on the operation of online roll call, interactive teaching materials, online quizzes, interactive tools, handing in homework, or other functions of the online teaching system adopted by the school. These seed teachers and the guidance of school teaching units can help reduce students’ uncertainty about receiving distance teaching in terms of trialability. Teachers can also directly give guidance to students who have problems with online operations during online teaching, whether in class discussions or online operation submissions and convey the correct usage methods to reduce students’ uncertainty in using online teaching software.

The results of this study show that perceived ease of use has a significant impact on perceived usefulness. Therefore, it is suggested that students take the initiative to learn how to use the online teaching system operations adopted by the school, such as the most basic exercises of how to switch screens from the microphone to the camera, how to operate on the screen for uploading assignments, and completing online discussions. They must be familiar with the operating interfaces. There are many synchronized video software related to distance teaching, and they must have a further understanding of the functions of the systems adopted by the school. This will reduce the frequency of reinstalling or resetting for every online class, make the operation of online teaching run smoother, and show the results of using the distance teaching system to improve the learning effectiveness.

The results of this study also show that the impact of perceived usefulness on attitude of use reaches a significant level. Therefore, this article recommended that students adapt to teaching and learning in the same “virtual space” as soon as possible and to interact with the teacher in real-time. It is different from physical teaching as both teachers and students now are having real-time two-way communication with the microphone and remote cameras. In the past, students have been accustomed to face-to-face communication with the teacher in the classroom, and when it is converted into another space, the teacher can see the students participating in the classroom, and the students can also see the teacher. Therefore, it is recommended that students learn to adapt to the new learning environment and thus obtain the nutrients of learning from distance teaching. The adaptation process of such a conversion in learning will help students to hold a positive attitude toward distance teaching and also make them happier to learn.

The results of this study also indicate that attitude of use has a significant level of impact on behavioral intentions. Therefore, it is suggested that students should have an understanding of the diversified learning styles under the epidemic. The students should understand that such learning styles can bring different experiences and challenges to themselves, while schools are obligated to provide teaching and learning of improved quality. Only when they have a positive attitude toward the online teaching system can they use the online teaching system more seriously.

It can be seen from the research results that nine research hypotheses in this article are not valid, and the standardized regression coefficient values are low. It is recommended in future that researchers use questionnaires, interviews, and on-site observations to extensively analyze the process of teachers using online teaching software. After further reviewing the problems commonly encountered by teachers, they can further describe the research hypotheses more rigorously and more precisely. For example, Hypothesis 10 of this study points out that related advantages have no significant impact on attitude of use. In other words, neither the difficulties encountered by teachers in the process of adopting distance teaching software nor the learning process of students was enough to make them think that distance teaching is more advantageous than traditional classroom teaching. Therefore, future related research can collect relevant information and corresponding solution strategies, and then research hypotheses that are more in line with the current situation of the education scene can be developed to offer practical suggestions after empirical analysis.

## Data availability statement

The original contributions presented in the study are included in the article/supplementary material, further inquiries can be directed to the corresponding author.

## Author contributions

All authors listed have made a substantial, direct, and intellectual contribution to the work and approved it for publication.

## Conflict of interest

The authors declare that the research was conducted in the absence of any commercial or financial relationships that could be construed as a potential conflict of interest.

## Publisher’s note

All claims expressed in this article are solely those of the authors and do not necessarily represent those of their affiliated organizations, or those of the publisher, the editors and the reviewers. Any product that may be evaluated in this article, or claim that may be made by its manufacturer, is not guaranteed or endorsed by the publisher.
